# Comparison between Acid Digestion (ICP-OES) and X-ray Fluorescence (XRF) Spectrometry for Zinc Concentration Determination in Rice (*Oryza sativa* L.)

**DOI:** 10.3390/foods12051044

**Published:** 2023-03-01

**Authors:** Sharmin Sultana, Husne Ara Khatun, Muhiuddin Faruquee, Md Mizan Ul Islam, Hosna Jannat Tonny, Md Rafiqul Islam

**Affiliations:** IRRI-PPP Grain Quality Testing Laboratory, International Rice Research Institute, Dhaka 1213, Bangladesh

**Keywords:** X-ray fluorescence spectrometry, ICP-OES, rice, zinc, elements

## Abstract

The determination of mineral concentrations in rice grain samples is crucial for analyzing their nutritional content. Most mineral content analysis techniques depend on inductively coupled plasma (ICP) spectrometry and are often complicated, expensive, time-consuming, and laborious. Recently, the handheld X-ray fluorescence (XRF) spectrometer has been randomly used in earth sciences; however, it is hardly practiced in quantifying mineral content in rice samples. In this research, the reliability of XRF results was compared with that of the ICP-OES to determine zinc (Zn) concentration in rice (*Oryza sativa* L.). Approximately 200 dehusked rice samples and four known high-Zn samples were analyzed using both XRF and ICP-OES techniques. The concentrations of Zn were recorded using the XRF technique and then correlated with the ICP-OES results. The results indicated a high positive relationship between two methods, with *R*^2^ = 0.83, *p* = 0.000, and the Pearson correlation value of 0.91 at the level of 0.05. This work demonstrates the potential of XRF as a reliable and low-cost as well as an alternative technique to ICP-OES methods for determining Zn content in rice as it allows the analysis of a greater number of samples in a short period at a considerably low price.

## 1. Introduction

Rice is a major cereal crop which is cultivated all over the world and feeds thousands of people globally. Rice is a relatively cheap source of nutrients, and demand is rising sharply, with 112 million metric tons of rice expected to be produced by 2035 to feed a growing global population [1]. In some parts of the world, rice consumption is as high as 990 g day^−1^ persons^−1^ [2]. Rice provides a livelihood for more than three billion people in Asian countries alone, underlining its importance not only as a human food but also as one of the most economical crop plants for research [3].

It is estimated that approximately 2 billion people are impacted by mineral deficiency; for instance, 60% of patients are anemic due to iron (Fe) deficiency and over 30% are anemic due to the deficiency of Zn [3,4]. More than half of the world’s population suffers from deficiencies of bioavailable nutrients and at least 50% of children worldwide suffer from micronutrient deficiencies [5], especially in the least developed countries where Zn is the most important micronutrient and the consequence of its deficiency causes reduced immunity, stunting, mental lethargy, lesions on the eyes and skin, delayed healing of wounds, and diarrhea [6]. Not surprisingly, in Asia, rice is considered life and serves as a vital primary source of energy; however, in countries where rice serves as a major staple crop, micronutrient deficiencies are the most prevalent there [7].

In developing countries, the demand for most of these micronutrients can be met through the direct consumption of cereal products. Among various cereal products, rice ranks second in dietary uses all over the world, and due to its use as a staple food, it can be utilized as a carrier for transporting micronutrients from plant sources to human bodies [3,7]. Nevertheless, like other cereal products, rice contains comparatively low levels of vital micronutrients such as Zn and Fe [2,8]. For instance, polished rice contains a small amount of Fe and Zn which can cover only 20% of the daily requirement of Zn and Fe for an individual [2,8]. Thus, the global prevalence of micronutrient deficiencies can be controlled by the introduction of fortification techniques or periodic supplementation [2,8]. Biofortification or periodic supplementation with target micronutrients is an effective way to engage communities which are at a higher level of health risks, especially infants, children, and women [2,5]. 

For the past few years, biofortification has appeared to be the best long-term strategy to prevent micronutrient deficiencies throughout the world [9]. Nonetheless, biofortification through plant breeding strategies often requires screening several potential germplasms in the initial stage (advanced breeding lines) and deploying them further in inbred or hybrid varietal development programs. Thus, physiological studies regarding the micronutrient translocation in varied stages of plant growth, as well as a final polished rice grain, are needed [9]. 

To determine micronutrient concentration accurately, tools that are less laborious with a low price, high precision, accuracy, and high throughput are essential for routine use. At present, the mineral content of rice grain can be measured with various techniques such as ICP-OES or atomic absorption spectroscopy (AAS). The principle of these techniques usually involves an emission spectrometric determination followed by acid digestion of the sample, and then the element emits energy at a specific wavelength specific to its chemical nature [9]. These techniques can identify and quantify the mineral concentration present in the rice grain and are often used in micronutrient analysis studies due to the high accuracy, reproducibility, and high-throughput nature of these techniques [9]. However, these techniques usually require high-purity samples and well-trained lab technicians. Additionally, these techniques involve lengthy sample preparation and thus incur more costs [9]. Similarly, particle-induced X-ray emission (PIXE) is another powerful technique to detect the micronutrients present in rice grains [10]. 

XRF and PIXE spectrometry are two well-known non-destructive techniques used for compositional analysis of constituents such as solid, thin films, as well as powder samples [11]. The principle of these methods is to excite the atoms of the substance to be investigated, and then elements are identified by the wavelengths of the emitted X-rays and the amount is determined by the intensity of those X-rays [11]. This technique has appeared as an effective and powerful technique for trace element determination from a wide source of samples including environment, biology, earth science, and forensic and medical science [10,11,12,13]. Hence, compared to the standard techniques, such as ICP-OES or AAS, XRF offers a low cost, high throughput, multi-elemental, and little or no sample preparation [9] (Table 1).

The development of portable XRF techniques has made it easier to quickly assess trace elements such as zinc and iron in rice grain [12,13]. XRF spectrometry is being broadly used for the quantitative assessment of biological samples, including their mineral content in advanced research laboratories [9,14,15,16,17]. Previously, ICP-OES was used for routine micronutrient quantification; nevertheless, the prolonged time for test accomplishment was one of the negative points of this techniques. Thus, recently, researchers have tried to establish XRF techniques as a possible replacement technique for ICP-OES, and previous literature indicated that there is a good correlation between the data analyzed via XRF and ICP-OES [13,18]. For instance, Khan et al. [13] documented the possibilities of using portable XRF as an alternative to ICP-OES for micronutrient analysis including Zn in barley grain with a short time period and low cost. They also found the concentrations of Zn from barley grain using an XRF analyzer and they correlated well with the ICP-MS results with a coefficient of *R*^2^ = 0.80. Similarly, Rouillon and Taylor [17] used an XRF analyzer to determine contaminated soils in some parts of Australia. The authors found a correlation between the sample data from XRF and data from ICP-AES for Mn, Pb, Cu, Zn, and Cd elements. A literature search revealed that XRF methods are suitable for elemental analysis, but no XRF analysis has been documented for the simultaneous quantification of micronutrient content in rice. To address this gap, we documented here the potential of XRF as an alternative technique to ICP-OES methods for determining Zn content in rice samples. 

**Table 1:** Comparison between XRF spectrometry and ICP-OES method.

Method Name	Key Features	Drawbacks	References
ICP-OES	ICP-OES is an emission spectrometric technique The major feature of this technique is each element emits energy at a wavelength specific to its chemical nature The intensity of the energy emitted at the specific wavelength is directly proportional to the concentration of the element available in the digested sample 1 g or 25–30 grains are required for a test	Destructive techniques Requires highly pure sample Continuous use of an argon flow is required to produce plasma Requires skilled research technicians Prolonged sample preparation Costly	[9,10,13]
XRF spectrometry	A famous technique for quantifying micronutrient content in plant materials The principle of this method is founded on the physical principle of emission of X-rays by a selected element Calibration using the known micronutrient standards is required Continuous use of an argon flow to produce plasma is not required Portable Non-destructive Highly accurate results Cost-effective Robustness of the results Minimum to no sample preparation required	Sample inhomogeneity is expected to be a major source of error as samples are analyzed as whole grains Large amounts (3–5 g) of samples are required Unable to analyze elements lighter than Na	[9,14,15,18]

## 2. Materials and Methods

### 2.1. Sample Collection

The IRRI grain quality testing laboratory (GQTL) in Bangladesh collected 200 advanced breeding line samples from the AGGRi Network Trials during the Aman season of 2021. Four known high-Zn rice samples, namely BRRI dhan 62, BRRI dhan 72, BRRI dhan 84, and BRRI dhan 100, were collected from the Bangladesh Rice Research Institute (BRRI) and used as a reference sample [19,20]. 

### 2.2. Preparation of Sample for XRF Spectrophotometer Analysis

A total of 100 g of well-dried paddy sample with a moisture content of 12–13% from each sample was dehusked using the PAZ-2/DTA Rice Miller dehusker (Zaccaria, Limeira, Brazil). The PAZ-2/DTA Rice Miller contains a roller made of a special polymer able to restrain Fe and Zn contamination. From the dehusked and cleaned rice samples, a sample of approximately 3–5 g was transferred to the sample cups and then covered with a thin clear plastic film. Afterward, the sample cups were gently shaken to achieve maximum sample homogeneity and then moved to the X-ray chamber. Prior to running the test, the analyzer head and sample were covered. Most of the XRF analyzers use a maximum of 40–50 kV excitation beams. In this study, we kept our experiment within 40 kV to enhance safety in our workplace for long periods of time. All measurements were taken with a protective X-ray chamber that completely eliminates excess X-ray beams that may scatter through the primary X-ray beam. Finally, to minimize error, XRF analyses were conducted in duplicate. The limit of detection and limit of quantification for XRF were calculated by the same method as described by Margui [21]. The moisture content of rice grain is an important factor for XRF analysis. Therefore, we kept the average moisture content of our tested sample in the range of 11–13%. Similarly, sample inhomogeneity was expected to be a major source of error as samples were analyzed as whole grains; therefore, two replications of sample data were recorded to eliminate error.

### 2.3. Preparation of Sample for ICP-OES

From each variety, 25–30 dehusked rice grains were selected. First, the samples were digested in a silica gel desiccator for approximately 24 h, mixed manually, and subsequently ground and sieved to ensure sample homogeneity with a 200-mesh particle diameter (75 µm). Afterwards, 0.25 g of the sample was digested by the CEM Mar X Microwave digestion system (CEM Crop., Matthews, NC, USA). The digested sample was then filtered and stored in plastic vials until use by ICP-OES (Perkin Elmer Inc., Sheldon, CT, USA). The wavelength for the elemental analysis of Zn was 213.857 nm. The limit of detection and limit of quantification were determined by the same method as described by Margui.

The absorption quantity of element increases with the increase in pH [10]. It has been documented that the highest extraction (up to 98%) of the analytes is found in the pH range of 4–6 [10]. Therefore, a pH of 5 was chosen as the optimum pH for this study.

### 2.4. Statistical Analysis

All measurements were performed on the same sample in triplicate, and the results were reported. The correlation between XRF and ICP-OES reference values was analyzed using a simple linear regression model. Pearson correlation, standard deviation, and standard error were calculated, as well as correlations between XRF and ICP-OES reference values using SPSS 20.0 at a 95% significance level. The variability in each category of Zn estimation was represented as box plots using R software. 

### 2.5. Method Calibration

XRF spectrophotometry requires an initial method of calibration using the known micronutrient standards as well as a blank sample. Four known high-Zn rice samples, namely BRRI dhan62, BRRI dhan72, BRRI dhan84, and BRRI dhan100, were used as a reference calibration standard for the XRF spectrophotometer. The raw XRF data were calibrated by linear regression equations defined by plotting measured values against the results of four known high-Zn materials. The same reference materials were also tested with ICP-OES. The results are tabulated in Table 2. Surprisingly, the zinc value of known reference materials was much lower in ICP-OES than in their published reports (Table 2). 

However, Zn values for the XRF spectrophotometer were like those published by BRRI for these four check varieties [19,20]. Thus, it can be concluded that the calibration standard for the XRF analyzer was strong enough and justified validation. 

## 3. Results and Discussion

The deficiency of micronutrients and vitamins in the human diet affects the human health of about two billion people in developing countries alone [21,22]. Among the essential micronutrients, Zn is one of the vital micronutrients for human beings and its deficiency may cause hidden hunger, which has been known as a serious human health problem as it may be associated with severe growth retardation, loss of appetite, and impaired immune function [2,22]. This problem could be minimized with the biofortification of one or two essential minerals in staple crops [22]. Biofortification technology of rice with essential micronutrients is an active approach for improving micronutrient status, especially in women and children [23,24]. Among the various staple crops, rice can act as a vehicle for the fortification of micronutrients, especially Fe and Zn, and thus could contribute promise for improving micronutrient status in the diets of rice-based communities [25]. Consequently, the identification of Zn concentration in breeding trials would be essential. ICP-OES and AAS are generally used for quantitative elemental analysis, yet the relatively long time for analysis accomplishment, which lasts about one week starting from sample collection, preparation, and end of the analysis, is one negative point of these techniques.

This research aims to demonstrate mineral content determination within the context of two technologies (XRF and ICP-OES) to facilitate breeding activities or biofortification programs. The estimation of the zinc concentration of rice is only indicative to establish the reliability of XRF. The high Zn concentration of biofortified rice was therefore tested by ICP-OES to confirm the XRF results.

Zn concentrations in rice samples of the advanced breeding lines, as measured by XRF and ICP-OES techniques, ranged from 12 ppm to 30 ppm and from 9.7 ppm to 26.2 ppm. The limit of detection (LOD) for the XRF analyzer was 3 ppm while that of ICP-OES was only 0.5 ppm. Similarly, the limit of quantification (LOQ) for the XRF analyzer was 10 while the LOQ for ICP-OES was 1.2. 

**Figure 1:**
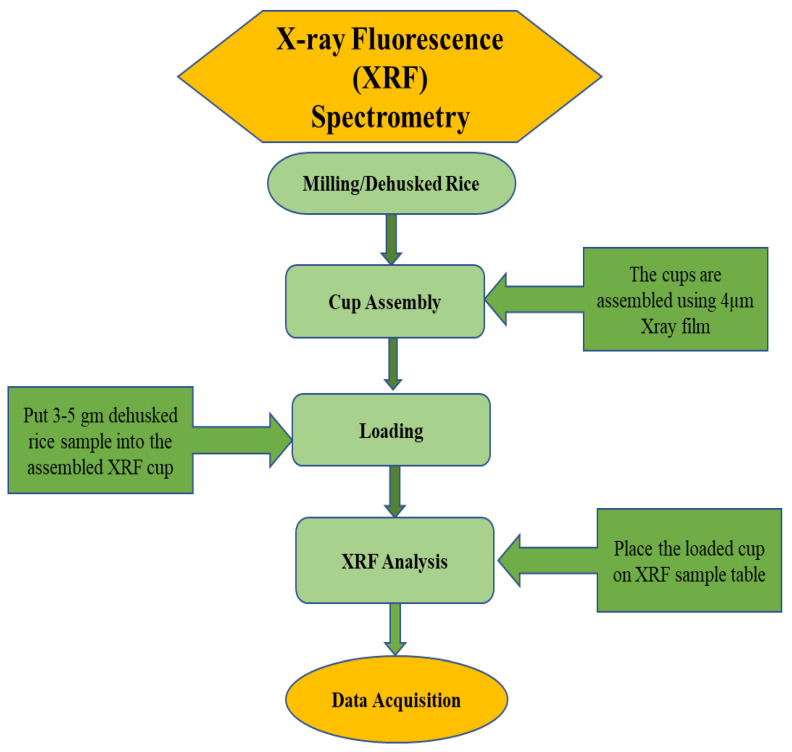
Detailed workflow of XRF analyzer for rice sample analysis.

Thus, the amount of Zn concentration using XRF technology is comparatively higher than that of ICP-OES for the tested 200 samples. The mean value of the XRF result of advanced breeding lines was less than 3.27 ppm compared with that of ICP-OES results in Figure 1. To validate the reliability of the XRF results, the data analysis of the Zn concentration of XRF and ICP-OES was performed, and we confirmed the results with the published data in Table 2 [19,20].

**Figure 2:**
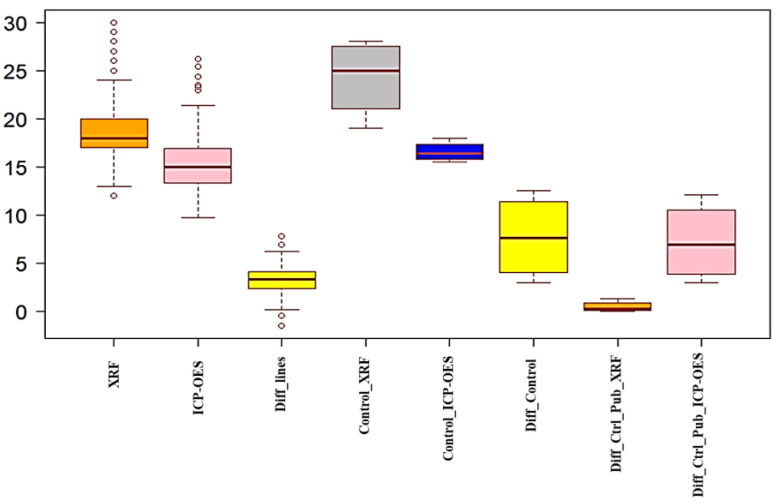
Mean difference in the Zn content of rice breeding lines and control reference sample BRRI dhan using non-destructive XRF analysis and ICP-OES.

According to the results, the reference sample of BRRI dhan had a mean difference between XRF and ICP-OES of 7.7 ppm (Figure 2). Furthermore, the mean difference in the Zn concentration of BRRI dhan using XRF with reference published data was 0.48, whereas ICP-OES showed a difference of 7.23 ppm when compared to published data (Table A1). The reason behind the high mean difference in Zn concentration between these two techniques may be due to the monitor indicators of ICP-OES analysis to detect contamination, which is not possible with XRF [18,26,27]. 

**Figure 3:**
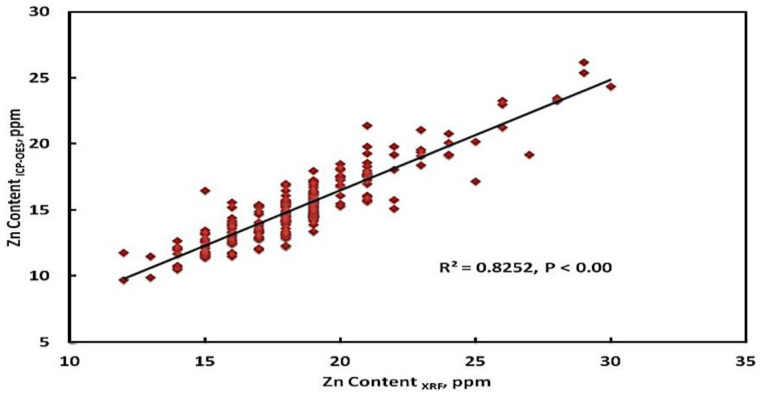
The correlation coefficient between the Zn content of rice cultivars was determined by XRF and by ICP-OES.

The correlation coefficient is a statistical measure of how strong a relationship is between two variables’ relative movements. Figure 3 depicts the correlation coefficient between Zn concentrations using XRF and ICP-OES technology. The results indicated a very high positive relationship between the Zn calibration method, with *R*^2^ = 0.83, *p* = 0.000, and the Pearson correlation value of 0.91 at the 0.05 level. A significant association of grain Zn contents with *R*^2^ = 0.83 has also been reported by other researchers; for instance, Khan et al. [13] found the concentrations of Zn from barley grain using the same method and they correlated well with the ICP-MS results with a coefficient of *R*^2^ = 0.80. Similar findings were also observed in the recent results for wheat [14,28], rice, pearl millet [15], potato [26], sorghum [27], maize, and bean [28] Zn determination using an XRF analyzer. 

Over the years, XRF technology has been used in several arenas, for instance, environmental remediation [29], petrology [30], geology [31], forensics [32], agriculture [33], and even mineralogy [34]. It is clearly indicated from this research that micronutrient analysis using XRF techniques is possible, and this technique will help researchers in investigating high-Zn content rice at the early stage of a breeding program, as it offers low-cost and rapid analysis. It has been documented that the genetic control of Zn content is linked, or that physiological mechanisms for the uptake or accumulation of Zn in the grains are interconnected. A significant positive association in grain Zn can result in simultaneous genetic improvement for micronutrients. 

**Figure 4:**
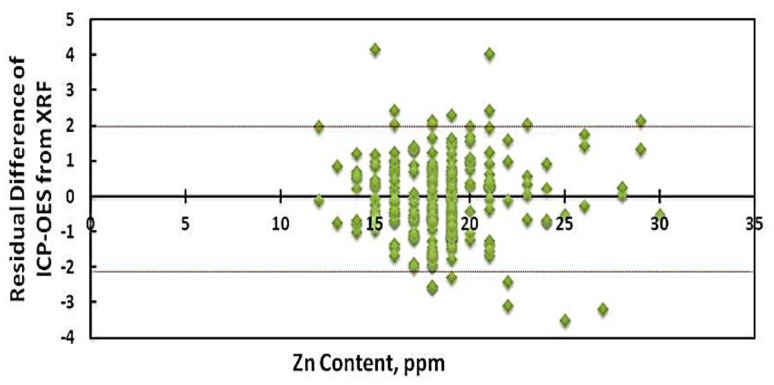
Residual difference in predicted Zn concentration of ICP-OES over XRF of 200 advanced breeding rice lines.

The regression model was used to verify the accuracy of the XRD result over ICP-OES for the data of 200 rice samples; Figure 4 demonstrates that the advanced rice cultivar line’s predicted Zn concentration of ICP-OES over XRF was nearly within the range −2 < Y > 2 and the number of interval deviants was acceptable. Paltridge et al. [15] reported the result of Zn concentration in rice grain and revealed that EDXRF predicted Zn in rice to within ±1.9 mg kg^−1^ of ICP-OES values, at a 95% confidence level, which is similar to the findings of XRF and ICP-OES (Table A2). These results demonstrate that XRF techniques are a straightforward and precise method for measuring the mineral profile of rice varieties.

## 4. Conclusions

Many plant breeding initiatives have focused on the production of high-mineral-content rice to improve human nutrition by correcting mineral disorders. This has resulted in a large increase in Zn levels in basic food crops. Rice cultivars with high-micronutrient-content grains could help to alleviate widespread deficits of the micronutrient in people who rely significantly on rice for their micronutrient needs and major dietary energy. It is noteworthy to indicate that the breeding of high-mineral-content rice must not compromise farmer-preferred traits as well as grain yield. Thus, fast screening of essential mineral content in breeding trials is imperative, but these analyses are not easy to implement in the initial stage of a breeding program due to the higher cost. Compared to the existing techniques (ICP-OES), XRF is a competent and cost-effective technique for testing a large number of materials in the initial stage of breeding programs. It can be concluded from the recent study that XRF is capable of generating reliable, rapid, and high-quality micronutrient analysis data. The added benefits of reduced operating costs with minimal sample and consumable preparation together with the ease of operation and minimal laboratory requirements make the XRF spectrophotometer an ideal technique for screening high-micronutrient crops on-site without requiring highly specialized laboratories and staff. 

## Figures and Tables

**Table 2:** Zinc value of reference materials.

Reference Varieties	XRF Value (mg/Kg)	ICP-OES Value (mg/Kg)	BRRI Published Value (mg/Kg) [19,20]
BRRI dhan62	19	16	19
BRRI dhan72	23	18	22.8
BRRI dhan84	28	15.5	27.6
BRRI dhan100	27	16.7	25.7

## Data Availability

Data is contained within the article.

## Appendix A

**Table A1:** Descriptive statistics of 204 advanced breeding lines.

Method	Max	Mean	Min	SD	SE
**XRF**	30	18.63	12	3.12	0.22
**ICP-OES**	26.2	15.36	9.7	2.88	0.20

Max = maximum, Min = minimum, SD = standard deviation, SE = standard error.

**Table A2:** Analysis of variance (ANOVA).

Source of Variation	SS	df	MS	F	*p*-Value	F-Crit
**Between groups**	1083.01	1	1083.01	119.58	0.00	3.86
**Within groups**	3659.06	404	9.06			
**Total**	4742.07	405				

SS = sum of squares, df = degrees of freedom, MS = mean sum of square, F-crit = F critical value.
